# Avoided wildfire impact modeling with counterfactual probabilistic analysis

**DOI:** 10.3389/ffgc.2023.1266413

**Published:** 2023-11-08

**Authors:** Matthew P. Thompson, John F. Carriger

**Affiliations:** 1 Human Dimensions Program, USDA Forest Service, Fort Collins, CO, United States,; 2 Office of Research and Development, US Environmental Protection Agency, Cincinnati, OH, United States

**Keywords:** simulation, risk, event attribution, climate, fuels management, mitigation, effectiveness

## Abstract

Assessing the effectiveness and measuring the performance of fuel treatments and other wildfire risk mitigation efforts are challenging endeavors. Perhaps the most complicated is quantifying avoided impacts. In this study, we show how probabilistic counterfactual analysis can help with performance evaluation. We borrow insights from the disaster risk mitigation and climate event attribution literature to illustrate a counterfactual framework and provide examples using ensemble wildfire simulations. Specifically, we reanalyze previously published fire simulation data from fire-prone landscapes in New Mexico, USA, and show applications for post-event analysis as well as pre-event evaluation of fuel treatment scenarios. This approach found that treated landscapes likely would have reduced fire risk compared to the untreated scenarios. To conclude, we offer ideas for future expansions in theory and methods.

## 1. Introduction

In the Western United States and elsewhere, the growing damages from wildfires lead to calls for increased investment in hazardous fuel reduction to protect the wildland–urban interface (WUI), among other objectives. Limited resources and operational constraints, among other factors, limit the areas where treatments can be implemented. Therefore, there is a clear need for strategic prioritization based on information about what fire and fuel management scenarios are most likely to be effective.

However, the informational basis regarding fuel treatment effectiveness is mixed. Empirical analyses have found that treatments can be effective at reducing fire intensity and severity within treated areas, but evidence of their effectiveness at landscape scales is limited (Fernandes, 2015; Kalies and Kent, 2016; McKinney et al., 2022; Ott et al., 2023; Urza et al., 2023). This gap is critical as many existing or proposed treatments are not directly located in the WUI but rather in proximal wildlands, based on the premise that treatments will interrupt fire spread and reduce WUI exposure. Simulation modeling is widely used to fill that gap, showing how treatments can affect metrics such as landscape burn probability (Ott et al., 2023). However, in almost all cases, simulation modeling is used to evaluate hypothetical future treatments (e.g., Benali et al., 2021; Alcasena et al., 2022), with few examples that analyze how prior treatment interactions with wildfires may have altered landscape outcomes (e.g., Cheney, 2010; Cochrane et al., 2012). As a result, it can be difficult to highlight successful risk reduction interventions and quantify the historical return on investment.

In this study, motivated by research in disaster risk reduction and climate science, we outline a potential pathway for estimating avoided wildfire impacts that blends counterfactual probabilistic modeling with extreme event attribution. Counterfactual reasoning allows events that were not realized to be examined. Large wildfires have numerous risk factors due to both climate change and on-the-ground fuel conditions. Understanding whether a wildfire would or would not have exceeded thresholds for extremes based on these antecedents can help examine the influence of key variables on wildfire outcomes. Counterfactual analyses take into consideration the probability of an event (i.e., a fire of a particular size) occurring in an observed world and an imaginary world where causes are present or absent, unlike the real one (Lott and Stott, 2016).

One central idea is that modeling unrealized events in the absence of mitigation interventions can shed light on the often hidden benefits of risk reduction programs (Lin et al., 2020; Rabonza et al., 2022); that is, successful interventions can be made invisible by their very success, which could reduce awareness or continued investment. Importantly, this shifts the baseline for evaluation from realized outcomes to averted outcomes and characterizes management efficacy in risk-based terms.

Another central idea is that event attribution logic can provide a common language and framework for probabilistic analysis of counterfactual events. Typical applications examine the influence of climate change in extreme weather events, where the counterfactual scenarios experience less severe events due to the absence of climate forcing (Hannart et al., 2016; Otto, 2017; Naveau et al., 2020). Several studies have used these methods to attribute growing wildfire hazards to climate change (Kirchmeier-Young et al., 2017, 2019; Tan et al., 2018; Van Oldenborgh et al., 2021). In this study, we modify the event attribution framework to consider counterfactual scenarios without risk reduction interventions where the unrealized wildfire outcomes could have been more severe, as well as hypothetical scenarios where the current landscape conditions exhibit exacerbated hazards due to a legacy of fire exclusion and forest management practices and could experience more severe outcomes relative to treated conditions.

To demonstrate the key concepts, we reanalyze previously published results from two simulation studies that modeled large fire growth on landscapes in New Mexico, USA (Thompson et al., 2016, 2022). These studies address salient fire and fuel management challenges in fire-prone forests, simulate fire on counterfactual untreated and hypothetically treated landscapes, and reanalyze modeling results in a stylized manner for illustrative purposes. Note that all relevant fire size data are either provided in this study or are available in online data archives (Vogler et al., 2022). We first review the basic framework, then present applications of landscape scenario evaluation for post-event counterfactual analysis and pre-event hypothetical analysis, and finally, discuss ideas for future expansions in theory and methods.

## 2. Materials and methods

### 2.1. Basic framework

In this study, we provide a simplified overview of event attribution (Hannart et al., 2016) and pattern our analysis after a related example attributing extreme fire risk in Canada to climate change (Kirchmeier-Young et al., 2017). The first step is to define the characteristics and magnitude of the extreme event of interest, which here is a final fire size exceeding a specified threshold. More refined analyses in future could identify a richer set of assessment endpoints, for example, population that is exposed or area of critical habitat burned.

Two key event attribution metrics are the fraction of attributable risk (FAR) and relative risk (RR). Both are derived here by calculating fire size exceedance probabilities (i.e., probability of exceeding specified fire size thresholds) on the actual landscape (p0) and the counterfactual landscape (p1). FAR (Eq. 1) is the fractional likelihood of an event that can be attributed to external forces. RR (Eq. 2) is a measure of how many times more likely the event occurrence is on the counterfactual landscape.


(1) $$FAR=1-\frac{p_{0}}{p_{1}}$$

(2) $$RR=\frac{p_{1}}{p_{0}}$$

The FAR value interprets the potential influence of the antecedent to the event of interest by its closeness to 1. A FAR of 1 indicates that the event of interest is extremely unlikely to occur without the occurrence of the antecedent (Funk et al., 2019), though other antecedents can still cause the event to occur (Herring et al., 2018). A FAR of 0.5 indicates a doubling of the probability of the event occurring, while a FAR of 0 indicates no relationship (Burger et al., 2020). The RR provides a “signal-to-noise-ratio” metric of the risk factor that differs between the counterfactual and factual realms (Knutson et al., 2014). An RR of 1 indicates identical probabilities between the two groups and no differentiation. An RR > or <1 indicates increased or decreased risk, respectively.

### 2.2. Counterfactual analysis of past treatment and disturbance

In the first example, we rely on simulation data from Thompson et al. (2016), who modeled how the 2011 Las Conchas Fire, New Mexico, USA, could have grown on a counterfactual landscape without previously treated or burned areas (Table 1). Using the FARSITE modeling system (Finney, 2004), 10 instances of the Las Conchas Fire were simulated on both actual and counterfactual landscapes to account for stochasticity induced by the varying numbers, locations, and ignition probability of spot fires. At the time, only performing 10 simulations was deemed an acceptable tradeoff considering computing demands; more recent applications, as shown in Section 2.3, simulate many more fire events.

**FIGURE 1:**
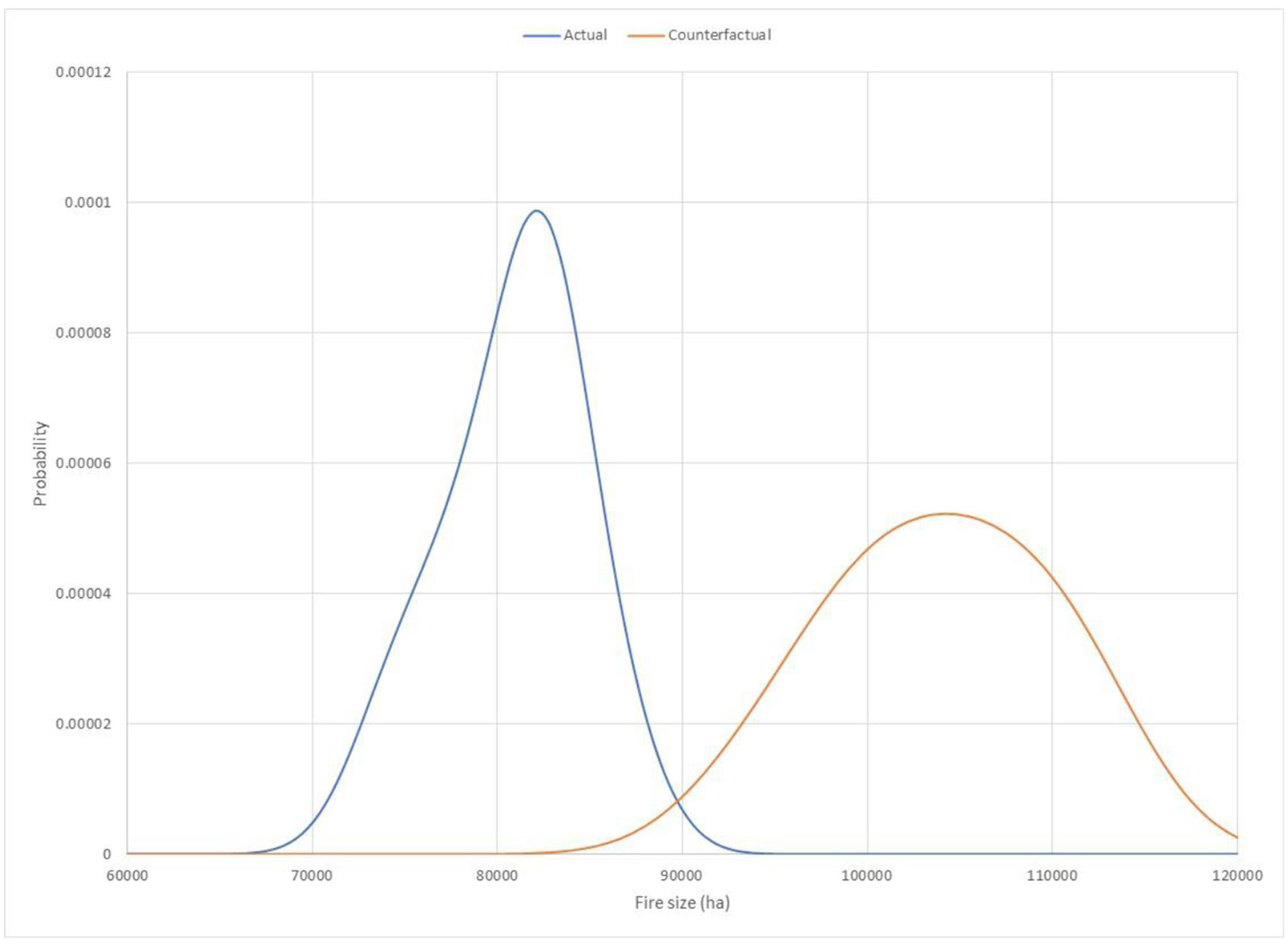
Density plots for the final fire size (ha) of the Las Conchas Fire, for the actual landscape in blue and the counterfactual landscape in orange. The blue curve corresponds to a probability distribution for the potential final fire size for the Las Conchas Fire (considering stochasticity in factors such as spotting) on the actual landscape burned by the Las Conchas Fire. The orange curve corresponds to a probability distribution for the potential final fire size of the same fire igniting on the same date and burning under the same weather conditions, but on a counterfactual landscape without the influence of previously treated and burned areas. Plots derived from simulation data in Table 1 using a Gaussian kernel density estimator.

Following the example of Kirchmeier-Young et al. (2017), we used simulation results to derive density plots using kernel density estimators (Figure 1) and empirically integrated the density curves to generate exceedance probabilities p0 and p1. In this study, p0 is the probability of exceeding a fire size threshold on the actual landscape burned by the Las Conchas Fire, and p1 corresponds to the counterfactual landscape without prior treatment or disturbance. For illustration, we defined an event threshold of 86,000 ha, which corresponds to a 90th percentile event on the actual landscape, or an exceedance probability of 0.10.

### 2.3. Hypothetical analysis of future treatment scenarios

We further illustrate the application of the FAR and RR metrics to the prospective evaluation of alternative treatment scenarios. In this case, we reanalyzed fire simulation data (Thompson et al., 2022; Vogler et al., 2022) that generated multiple hypothetical treatment scenarios according to distinct treatment prioritization schemes and variable treatment extents and compared performance across strategies for an 8.5 million ha case study landscape in New Mexico, USA. Relative to the earlier study on the Las Conchas Fire that sought to replay a single event, here, the simulation approach aimed to account for a broader set of stochastic elements related to the frequency, location, and timing of ignitions along with variability in fire weather, resulting in tens of thousands of simulated events using the FSim system (Finney et al., 2011). In this specific instance, over 89,000 unique fire events were simulated on the actual landscape.

Furthermore, because this analysis is forward-looking, there is no backward-looking counterfactual landscape to analyze, but rather only hypothetically treated landscapes. Therefore, that requires that we redefine p0 to be the exceedance probability for the hypothetically treated landscape and p1 to be the exceedance probability for the actual (untreated) landscape. In this case, the risk becomes an attributable or relative benefit from treatment extent. To simplify the presentation, we compare two hypothetical treatment scenarios against the baseline current condition (untreated) landscape. Because of our focus here on final fire size, we abstract away from the specifics of spatial prioritization schemes and instead isolate differences in treatment extent by comparing random scenarios that treat 5% (RAND5) and 25% (RAND25) of the analysis landscape.

## 3. Results

### 3.1. Counterfactual analysis

**FIGURE 2:**
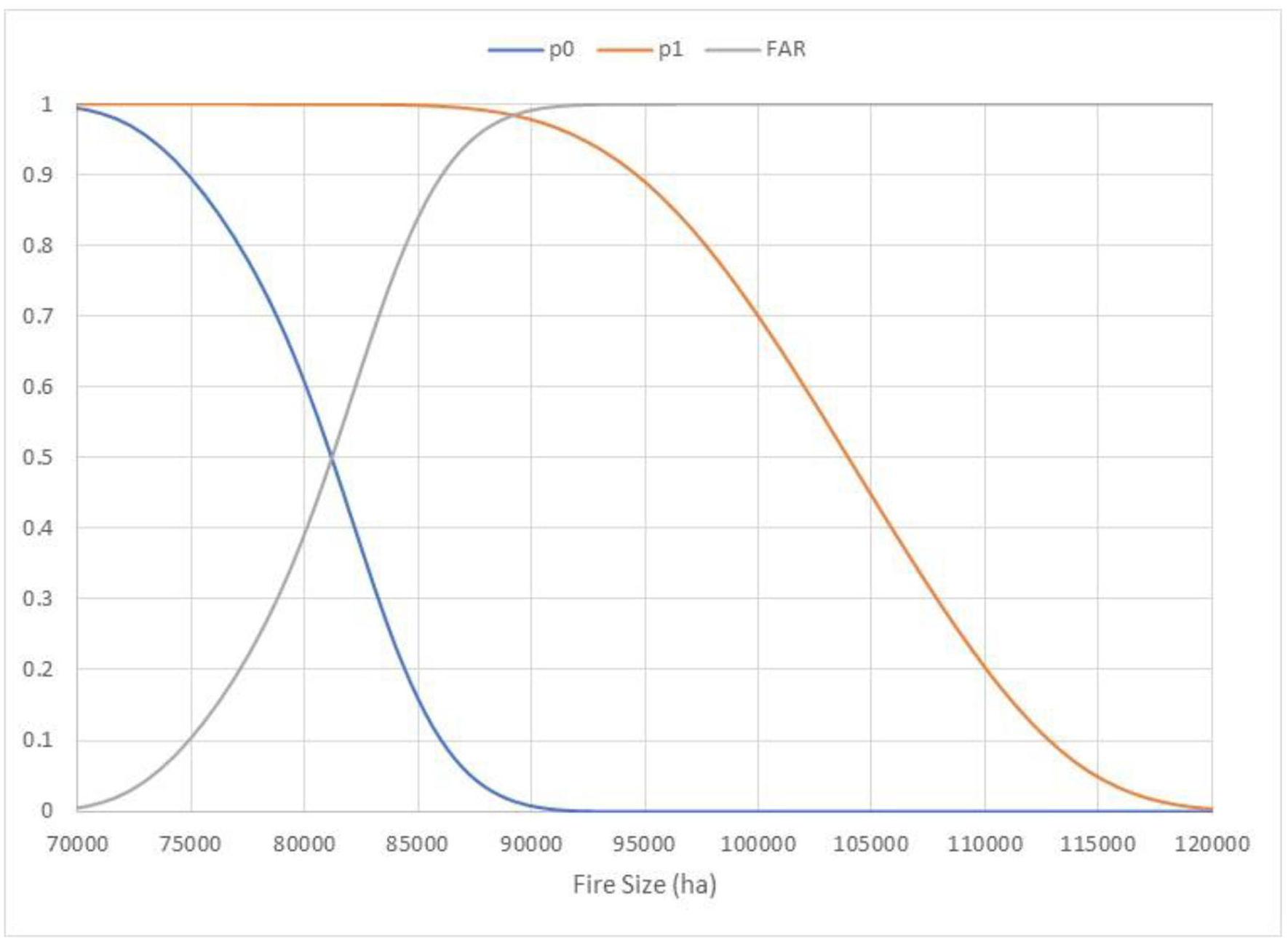
Plots of p0, p1, and a fraction of attributable risk (FAR; y-axis) for the final fire size of the Las Conchas Fire on the actual, treated landscape (p0) and the counterfactual, untreated landscape (p1). The exceedance probabilities are determined by empirically integrating the density curves shown in Figure 1; see Kirchmeier-Young et al. (2017). The probability of exceeding any given size threshold on the counterfactual landscape is always greater than or equal to the corresponding exceedance probability on the actual landscape. The exceedance probability curve on the actual landscape drops to near zero approximately a size threshold of 90,000 ha. As a result, the FAR curve rises steeply and approaches a value of ~1.0 this same size threshold.

Plots of p0, p1, and FAR for the Las Conchas Fire indicate that for the counterfactual landscape, the final fire size was likely to be much larger (Figure 2). Approximately 81,450 ha were burned at the 50th percentile on the actual landscape, while approximately 104,200 ha were estimated to be burned at the 50th percentile on the counterfactual landscape. In fact, there is little overlap on the exceedance probability curves; a fire size of 90,000 ha corresponds to an exceedance probability of 0.01 on the actual landscape but 0.99 on the counterfactual landscape. Because of this, the FAR curve rises steeply and approaches 1.00 approximately the same size threshold. The FAR also crosses 0.5 at 81,450 ha, indicating the risk of a 50th percentile fire is double for an untreated landscape.

**FIGURE 3:**
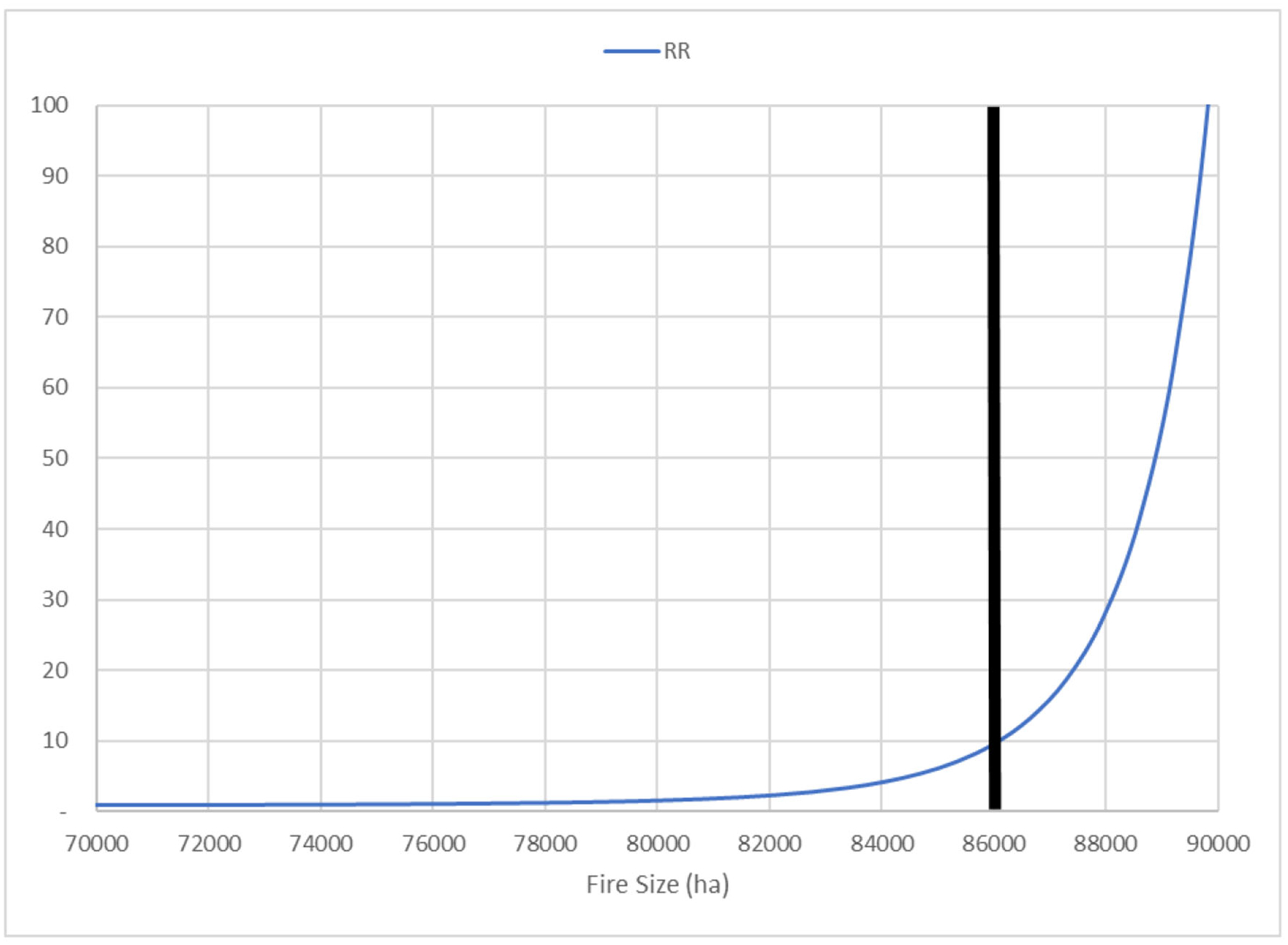
Plot of relative risk (RR; y-axis) for the final fire size of the Las Conchas Fire, with a threshold of 86,000 ha identified. This threshold corresponds to a 90th-percentile event on the actual landscape or an exceedance probability of 0.10 (see Figure 2). Here, RR indicates how many times more likely the Las Conchas Fire could grow to exceed 86,000 ha on a counterfactual landscape without previously burned and treated areas. The curve shows a fire of this size is 10 times more likely on the counterfactual landscape, and RR increases steeply beyond this threshold.

The RR for the Las Conchas Fire was ≥ 1.00 for all size thresholds (Figure 3). At the selected threshold of 86,000 ha, RR is approximately 10.00, implying that a fire of this size is 10 times more likely in the landscape without prior fuel treatment. Beyond that threshold, RR values rise steeply and approach 100, implying up to 100 times greater probabilities of occurrence for the larger fire sizes considered in the counterfactual landscape without previous fuel treatment. This also implies two orders of magnitude difference in probabilities of occurrence for a fire size exceeding 90,000 ha on the counterfactual landscape.

### 3.2. Hypothetical analysis

**FIGURE 4:**
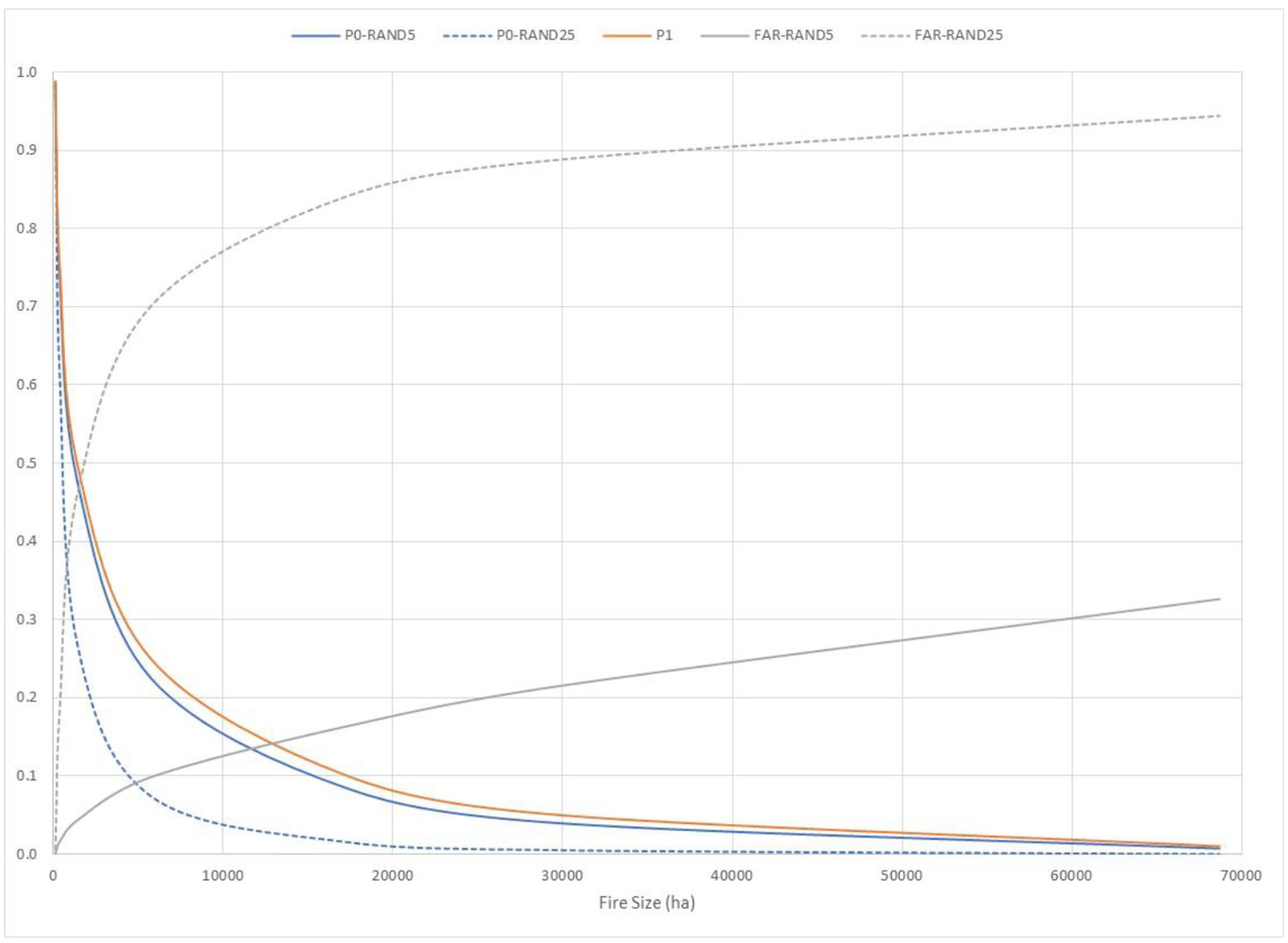
Plots of p0, p1, and a fraction of attributable risk (FAR; y-axis) for final fire size across thousands of simulated fire events, comparing two hypothetical treatment scenarios that locate treatments randomly at extents of 5% (RAND5) and 25% (RAND25) of the analysis landscape. In contrast to Figure 2, p0 corresponds to the hypothetical, *treated* landscapes and p1 corresponds to the actual, *untreated* landscape. Note that the exceedance probability curve for the actual, untreated landscape (p1) is always greater than or equal to the exceedance probability curves for the hypothetical landscapes, reflecting greater fire growth potential due to accumulated fuels. The difference in exceedance probabilities p0 and p1 is greater for the RAND25 hypothetical landscape, leading to higher values of FAR.

**FIGURE 5:**
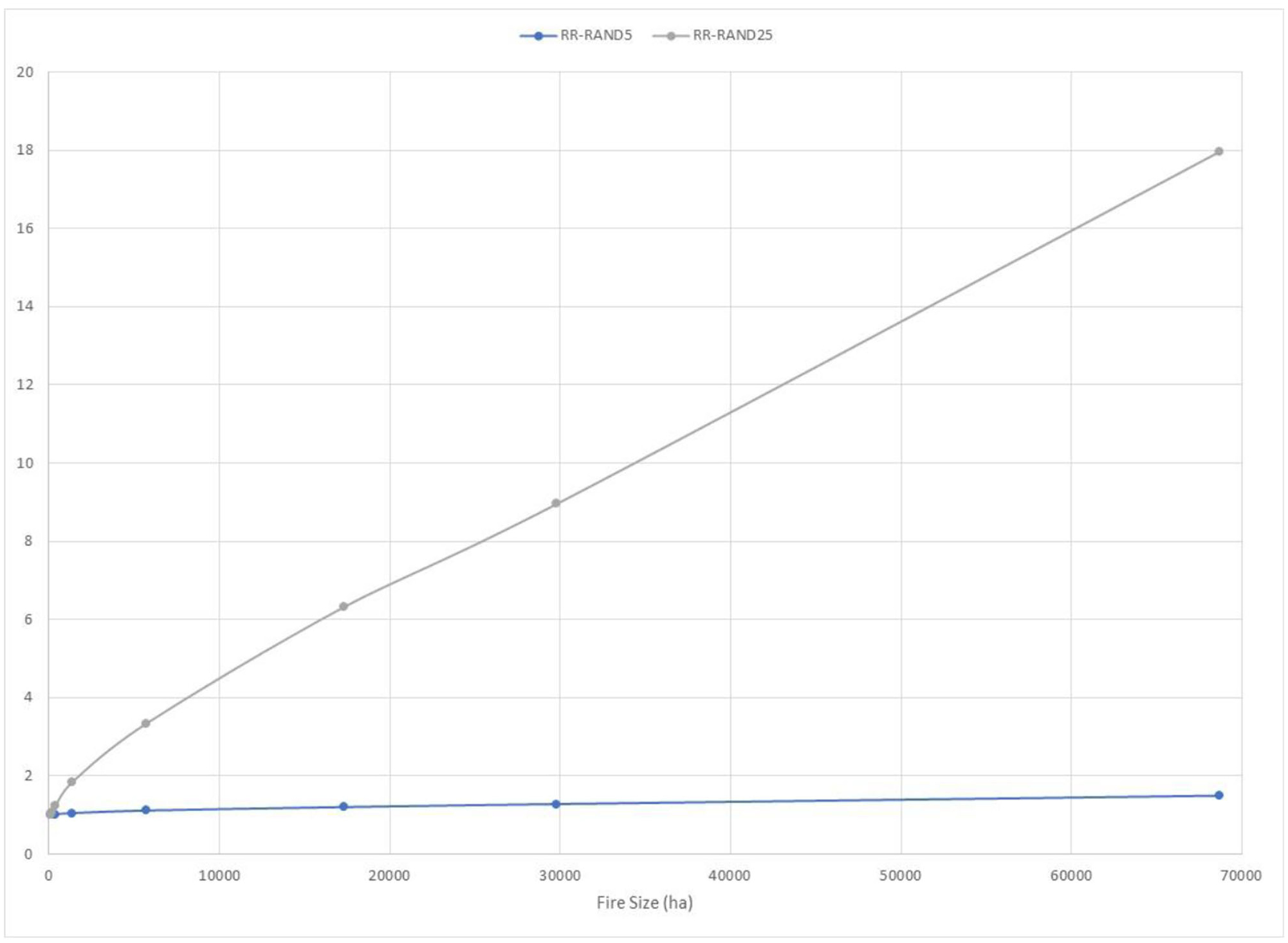
Plot of relative risk (RR; y-axis) for final fire size across tens of thousands of simulated fire events, comparing two hypothetical treatment scenarios that locate treatments randomly at extents of 5% (RAND5) and 25% (RAND25) of the analysis landscape. As fire size increases, RR values for the RAND5 landscape approach are only 1.5, whereas RR values for the RAND25 landscape approach are 18.0.

The p0, p1, and FAR for thousands of simulated potential fire events on the New Mexico landscape were calculated with hypothetical treatment scenarios (Figure 4). Relative to the Las Conchas example (Figures 2, 3), the use of p0 and p1 is reversed so that p0 is the counterfactual landscape receiving treatment and p1 is the factual landscape without treatment. The curves for p0 and p1 for these treatment scenarios drop much more steeply than the La Concha fire simulations (Figure 2), reflecting that this ensemble captures more fires burning under a broader range of conditions. FAR values increase with the fire size, and FAR approaches 1.00 for the larger treatment extent (RAND25). By contrast, for the RAND5 treatment scenario, FAR reaches approximately 0.33. The interpretation here is that the current condition of the landscape favors more extreme fire sizes relative to the hypothetically treated landscape. Moreover, FAR is lower on the 5% treatment landscape relative to a landscape with a 25% treatment extent due to greater differences in probabilities for fire sizes between the higher treatment regime and the untreated landscapes. Like FAR, RR increases with fire size, but grows much more steeply for the RAND25 scenario, reaching approximately 18.00 for fires exceeding 28,000 hectares (Figure 5). The interpretation is that a landscape treated randomly at 5% extent is not meaningfully different from the current condition landscape in terms of exceedance probabilities for extreme events, but treating larger extents begins to exhibit significant differences.

## 4. Discussion

The major contribution of this study is to introduce counterfactual probabilistic analysis and event attribution metrics for quantifying avoided outcomes and analyzing the historical performance of fuel treatments. The absence of such frameworks can perpetuate uncertainty about the return on investment from risk mitigation and lead to a reliance on anecdotes over quantitative metrics. Moreover, examination of the probabilities individually without a FAR or RR framework can lead to cognitive errors when interpreting the risks and benefits of fuel treatments. We illustrated how these event attribution metrics can be complementary to other simulation-based approaches to compare hypothetical treatment scenarios as well. One key reason to introduce the framework for prospective evaluation is to have a common language and logic for analyzing post-event and pre-event modeling exercises. In this process, attribution analysis should be conducive to localized hazards and their use in wildfire assessments.

Useful and interpretable explanatory tools for examining model outcomes can be derived from a counterfactual analysis (Kenny and Keane, 2021). The FAR and RR provide metrics for comparing the probabilities of events with or without antecedents. This was demonstrated in the interpretation and comparison of real-world and simulated outcomes for the two case studies here. Our reanalysis yielded FAR and RR values, suggesting a significantly higher potential for extreme fire growth on both actual and counterfactual untreated landscapes. Other case studies have used the RR and FAR to examine the influence of climate change variables on outcomes of interest, such as wildfires and heat waves. For example, two case studies in China used counterfactual RR analyses to clearly identify a more than doubling in probability for the occurrence of a heavy precipitation event like a record-setting one in 2017 (Sun et al., 2019) and heat waves as intense as the maximum observed index values in multiple regions from anthropogenic climate change influences (Sun et al., 2017). Although both the FAR and RR provide highly interpretable metrics in these and other studies, they may still be supplemented with other communication approaches that rely on more qualitative, context-relevant interpretations for communicating risks to varied audiences (Lloyd and Oreskes, 2018). In turn, these communication approaches, such as storylines and stakeholder co-developed modeling approaches, can assist in iteratively building attribution studies that are well-defined and useful in terms of potential risk factors and events.

The approach taken with the examination of RR and FAR across variable percentiles of fire size also illustrates how interpretations can be affected by how a fire size threshold is defined. Event definition is a major influence on the assessment of attribution (Lott and Stott, 2016). Single thresholds may provide a clear delineation but can be misleading if assessed alone. Large changes in the risk statistics were observed across distinct zones of fire sizes spanning tens of thousands of hectares, and much smaller changes were observed across much larger spans of fire sizes. Thus, understanding the implications across the full range of fire sizes would be useful in future applications. Hannart et al. (2016) also note the importance of the definition of the events to the outcome of counterfactual analyses for heat waves and climate change attribution.

The current case study assumed random treatments for demonstration purposes, but this may not be useful for all applications. Counterfactual analyses can also be useful for more complex modeling contexts. Future methodological explorations may include additional causal considerations in an attribution framework. Structural causal models help facilitate the identification and use of variables that can confound causal interpretations of risk metrics. Confounders are variables that causally influence both the impact and the treatment (Pearl and Mackenzie, 2018). In wildfire treatment, this may include variables related to fuel buildup that would causally influence the treatment locations and the wildfire magnitude. The depth of the relationships can also be expanded to account for cascading impacts through a multi-step attribution framework (Burger et al., 2020). The current study explored a single-step attribution framework that linked treatments to wildfires. However, a multi-step attribution framework may be useful for many applications (Burger et al., 2020). This would facilitate the evaluation of additional causal relationships between factors such as atmospheric climate change, fuels, and wildfires, along with resulting impacts on costs and benefits to human health, society, and the environment. There is often a causal web of influences that may need to be accounted for to understand the impacts of fuel treatments on wildfire outcomes. The development of structural causal models can provide the conceptual and quantitative basis useful for evaluating confounders in a multi-step attribution framework (Pearl and Mackenzie, 2018), necessitating a deeper examination of their potential in future research.

Future research may also examine how representing causal relationships can improve the interpretation and evaluation of counterfactuals. In addition to the metrics examined in this study, counterfactual analyses permit more detailed examinations of causality through probabilities of necessary, sufficient, and necessary and sufficient causation. The FAR is equated with necessary causation under certain assessment conditions (Hannart et al., 2016). However, utilizing the necessary causation over the FAR provides a more in-depth interpretation of the causal relationships between variables. Sufficient causality and necessary and sufficient causality, which were not addressed in this study, can capture additional conditions for causality not recognized by the FAR and RR metrics. Coupling attribution analysis calculations with causal Bayesian networks will allow greater complexity in metrics and compound events to be calculated (Carriger et al., 2021). In a causal framework, the counterfactuals provide a means for looking at how changing causal antecedents could have influenced or changed the observed effects and provide actionable insights.

Beyond advances in theory on the horizon, there are practical modeling considerations to address as well. We reanalyzed existing published data to illustrate the key points using fire size as our basis of comparison, but there is a clear need to expand the framework beyond area burned to other fire impacts. Furthermore, existing models may poorly capture the efficacy of fire suppression operations or how they are enhanced by landscape fuel management (Plucinski, 2019), which gets to the broader point that all modeling exercises are subject to issues of data quality and uncertainty. Caution is warranted when aiming to credibly recreate past fire events, and attention to detail on data quality control is essential. With that said, advances in remote sensing, geospatial, and computing technology provide greater opportunities for more refined analysis compared to what was performed for the Las Conchas Fire. Furthermore, given that agencies such as the USDA Forest Service prioritize investments in the order of billions of USD based on simulation models and hypothetical treatment scenarios, it stands to reason that the same family of models could be used to support analysis of the benefits of those investments in terms of outcomes from real interactions with real fires. We hope this article catalyzes additional interest and research into quantifying avoided wildfire impacts and revealing often unseen mitigation effectiveness.

## Figures and Tables

**TABLE 1:** Simulation results for the final fire size of the 2011 Las Conchas Fire on actual and counterfactual landscapes, sorted from smallest to largest.

Final fire sizes (ha) (Actual landscape)	Final fire sizes (ha) (Counterfactual landscape)
74,034	93,697
76,379	97,847
78,883	99,808
79,492	100,588
82,179	103,237
82,263	105,645
82,428	105,974
83,019	108,534
83,339	111,411
86,471	112,049

## Data Availability

The original contributions presented in the study are included in the article/supplementary material, further inquiries can be directed to the corresponding author.
